# Morphine Habit

**Published:** 1890-02

**Authors:** 


MORPHINE HABIT.—An efficient means of detecting the morphine habit is by adding a few drops of tincture of perchloride of iron to the patient's urine. A characteristic blue tinge results if he is a morphine user.—*N. Y. Medical Times.*

---

The following quaint epitaph on husband and wife—the husband having died first—is to be seen in a Parisian cemetery: "I am anxiously awaiting you.—A. D. 1827." "Here I am.—A. D. 1867." The good lady had taken her time about it.

